# Life under tension: the relevance of force on biological polymers

**DOI:** 10.52601/bpr.2023.230019

**Published:** 2024-02-29

**Authors:** Matthew T. J. Halma, Longfu Xu

**Affiliations:** 1 Department of Physics and Astronomy and LaserLab, Vrije Universiteit Amsterdam, De Boelelaan 1081, 1081 HV, Amsterdam, the Netherlands; 2 LUMICKS B. V., 1081 HV, Amsterdam, the Netherlands

**Keywords:** Tension, Optical tweezers, Molecular force

## Abstract

Optical tweezers have elucidated numerous biological processes, particularly by enabling the precise manipulation and measurement of tension. One question concerns the biological relevance of these experiments and the generalizability of these experiments to wider biological systems. Here, we categorize the applicability of the information garnered from optical tweezers in two distinct categories: the direct relevance of tension in biological systems, and what experiments under tension can tell us about biological systems, while these systems do not reach the same tension as the experiment, still, these artificial experimental systems reveal insights into the operations of biological machines and life processes.

## INTRODUCTION

The role of tension in biological systems is multifaceted, including force generation and sensing in a muscle sarcomere (ter Keurs *et al*. 1978), microtubule dynamics (Akiyoshi *et* *al*. 2010; Hamant *et al*. 2019), cell division (Curtis and Seehar 1978; Scarpa *et al*. 2018), processivity of molecular motors (polymerases (Goel *et al*. 2003; Wuite *et al*. 2000), helicases (Li *et al*. 2016; Ribeck and Saleh 2013), ribosomes (Harrington *et al*. 2020)), as well as regulating binding patterns (Skinner *et al*. 2011) and enzymatic processes (Wuite *et* *al*. 2000). These contexts can be studied in experimental systems, which prioritize studies of tension. As users and developers of optical tweezers (OT) instruments, we are left with the question: what is it exactly that we are measuring?

Clearly, the primary measurements include forces, distances, and torques, along with derived quantities such as energy. Furthermore, we can also manipulate the system on those parameters. Once we’ve established those as the basics, the next question may bluntly be stated: “Who cares?”. This question necessitates a deeper answer than merely the measurements being impressive from a technical perspective, which they undoubtedly are. The question then arises: Do biophysical measurements offer novel insights into life processes? We posit that indeed, they do, by uncovering the underlying mechanisms of biological phenomena.

The simple and boring way to cover this question is to state the specific applications: as mentioned above, these include, microtubule dynamics (Akiyoshi *et al*. 2010; Hamant *et al*. 2019), cell division (Curtis and Seehar 1978; Scarpa *et al*. 2018), processivity of molecular motors (polymerases (Goel *et al*. 2003; Wuite *et al*. 2000), helicases (Li *et al*. 2016; Ribeck and Saleh 2013), ribosomes (Harrington *et al*. 2020)), binding dependence on tension (Skinner *et al*. 2011) and tension-dependent enzymatic processes (Wuite *et al*. 2000). Really this is just a very narrow survey, and the applications are far beyond that.

The deeper way to answer the question would be to identify the qualities or preferably, singular quality, of biophysical experiments that differentiate them from the many other experiments that one could perform, and what unique value they offer. Whereas biochemistry elucidates the chemical facets of biological reactions; biophysics delves into the precise mechanisms of these processes, offering a unique window into the molecular dance of life. This approach has successfully provided insights into biological motors, describing the motions of their subunits and even being able to compute the respective forces and velocities.

What is unique about biophysics is that it at least approaches the most fundamental mechanistic understanding of biology. Biophysics is the study of the interface between physics and biology. Is the purpose of biophysics to explain the mechanisms of life? Most physics phenomena are described in terms of abiotic processes, which includes physics in general except for biophysics and certain high-level models of societies and economies. The resistance towards accepting biophysics stems largely from the association of physics with lifeless forces and energies. However, if we assume that the universe is governed by physical laws, it follows that life must also abide by these laws since it exists within our reality.

In this understanding, life can be seen as something special but not separate from the operations of the universe. This raises the issue of defining what exactly constitutes 'life,' which is a fascinating topic of discussion but one that we overlook for now. We begin with the most basic ways in which tension can manifest, in DNA molecules.

## FORCE RANGES IN BIOLOGY

Forces are crucial for cellular activities, although it is important to avoid the fallacy that understanding forces equates to understanding life. However, we can gain a detailed understanding of specific processes. By tracking force and position, we can discern the steps taken by molecular motors, for instance. Biophysics, when combined with a structural understanding, excels in this aspect. Knowing what is moving is valuable for developing therapeutics that interact with specific targets and identifying the protein components involved in various processes.

Additionally, force measurements can provide insights beyond the measured range of forces in a system. Critics often argue that optical tweezer measurements do not represent physiological tensions. While it is true that biological systems may experience tensions different from those reached in experiments, the point is acknowledged. This critique falls under the category of "artificiality", suggesting that the system fails to replicate meaningful biological phenomena, thus limiting the insights gained from such experiments.

Shifting our focus to generalizable insights obtained from tension experiments, we explore what perturbed systems can reveal about non-perturbed systems. One of the earliest applications of optical tweezers in biology aimed to answer questions about DNA behavior under high tension, causing structural transitions in the helix. The exact nature of this transition was initially unclear, with proposals ranging from helix straightening (B to S form transition), base peeling, to melting. Subsequent findings revealed that all three transitions could coexist depending on the geometry of the DNA handle to bead attachment (King *et al*. 2013).

So, what did this tell us about physiological DNA? First, let’s explore some other experiments, unfolding DNA hairpins. This work by Woodside *et al.* informed our understanding of the dynamics of base pair formation in nucleic acids (Woodside *et al*. 2006). Essentially, hairpins at tensions near equilibrium rapidly form and unform, dozens of times per second, which is not observable at bulk levels (Woodside *et al*. 2006). This informs a picture of DNA bases rapidly forming and dissociating, as opposed to the static picture one might have, largely based on the intuition of macroscopic objects.

Studying molecular motors interacting with nucleic acids adds another layer of complexity. For example, ribosomes have stalling forces of 13 ± 2 pN (Kaiser and Tinoco 2014), and polymerases have higher stalling forces of 20–35 pN (Forde *et al*. 2002; Wang *et* *al*. 1998; Wuite *et al*. 2000; Yin *et al*. 1995), above the unfolding force of strong hairpins (Woodside *et al*. 2006), and near that of some pseudoknots (Chen *et al*. 2009). For molecular motors, the force mechanics of the nucleic acid substrate can determine the subsequent mechanics of the molecular motor (Halma *et al*. 2019; Visscher 2016; Wuite *et al*. 2000). In the case of ribosomal frameshifting, this insight can be used in terms of therapeutics for viral illnesses (Kelly *et al*. 2021).

In the case of helicases, insight into the “movie” of what happens during duplex unwinding may be used in the development of helicase inhibitors, which can be useful in cancer therapeutics (O’Neil *et al*. 2017). We can combine force spectroscopy methods with structural studies to produce a coherent picture of physiological helicase action, as well as action that is impacted, due to a factor such as a mutation or an inhibitory compound.

Mechanobiology is the broad study of the impact of mechanical forces on biological systems, as well as the study of how biological systems exert forces. It is known for example, that one of the factors influencing cell differentiation in early embryogenesis is membrane tensional signals which can inform the cell if it is part of the ectoderm, mesoderm, or endoderm, and thus primes the development along a certain pathway of the epigenetic landscape.

## MEASURING TENSION

Before the advent of optical and magnetic tweezers, there was not a direct way of measuring tension on DNA. It was still possible to visualize cell division using microscopy, but any measurement of tension was inferred or extrapolated from other data. Then came the development of optical tweezers in 1978 by Ashkin which paved the way to discover the tensions operating on DNA strands.

**Figure 1:**
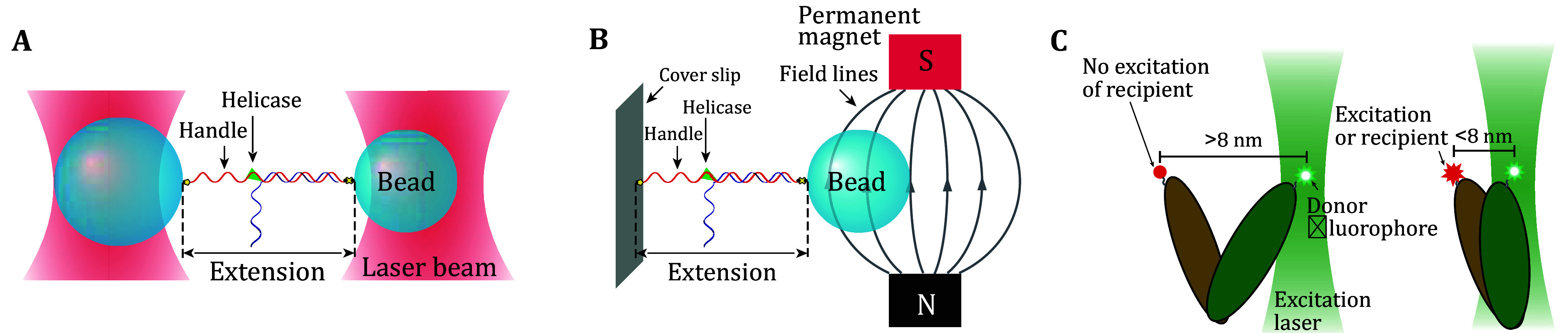
Single-molecule force-measurement methods. **A** Optical tweezers setup in studying a helicase protein. **B** Magnetic Tweezers setup. **C** FRET setup. A recipient fluorophore (red) emits a signal when it is within a threshold (shown here as 8 nm) distance from the donor (green)

Optical tweezers (OT) is a method capable of directly measuring biological forces at the single molecule level (Cost *et al*. 2015) (Fig. 1A). Unlike other techniques that observe for example membrane deformation and morphology, OT provides direct force measurements. Forces play a significant role in biology, where forces can induce conformational changes in molecules, acting as information stores.

Magnetic tweezers (MT) operate by a similar principle as OT, only the mechanism of controlling the bead position and field strength is through permanent magnets (Sarkar and Rybenkov 2016) (Fig. 1B). In specific setups, MT can also apply and measure torque on a bead.

Other ways of characterizing tension rely on measurements of distance, or of proxy force-dependent processes, such as binding of proteins. Other techniques include FRET to measure distances (Fig. 1C), and DNA origami techniques as calibrated force indicators (Cost *et* *al*. 2015). An additional possibility is using fluorescent protein binding rate as a ruler if these vary with DNA tension by a predictable relationship. One such example is Cas9, for which force increases off-target binding (Newton *et al*. 2019).

Beyond the above-mentioned techniques, there is a growing interest in alternative ways of generating tension, such as using acoustic (Sitters *et al*. 2015) or centrifugal force (Luo *et al*. 2023; Otake and Ukita 2019; Punnoose *et al*. 2020), which can offer new opportunities to explore biophysical questions. For instance, the non-invasive acoustic force can measure cell avidity without causing damage (Leick *et al*. 2022). One intriguing question that remains is whether there are other methods to generate and measure pico-to-nano level tension for studying molecular interactions.

### Tensioned polymers

#### Tensioned DNA/RNA

In the 1990s, experiments focused on polymer stretching experiments, essentially treating DNA as a polymer with relatively homogenous properties. One important discovery of this era was the discovery of the structural transitions undergone by DNA under tension. DNA when pulled, responds in four separate regimes (five if you count rupture). These are (1) the entropic regime, (2) the tension-response regime, (3) the overstretching transition, (4) the single-stranded regime, after which (5) the DNA can rupture, though this is usually due to the bead linkages, and not a break in the DNA itself.

The entropic regime occurs as the domains of the polymer get aligned, and this requires very minimal force, as the only resistant forces are largely hydrodynamic and entropic. This can be analogized to pulling on a pile of disordered strings from two ends. The string is not yet taught until the distance between the two ends approaches the contour length of the string. The dominant parameter of this regime is the persistence length L_p_. Once the string is taut, we have entered the tension regime, whereby the force-length relationship is dominated by the elastic modulus *K* of the material (DNA in our case). At approximately 65 pN, the regimes change to the overstretching transition, where strand peeling occurs, depending on the pulling geometry employed (King *et al*. 2013). The overstretching transition is marked by strand peeling, by the transition of B- (helical) to S-form (non-helical, like a straight ladder) DNA, and by the formation of melting bubbles. After the strands are completely unpeeled, a single strand of DNA connects the two beads, and the polymer behaves as a single strand of DNA is expected to behave. Increasing the tension further results in a rupture, though this is typically not a break in the DNA backbone, but instead either an inability for the trap restoring force to hold a bead, or the linkage of the DNA to the bead (usually biotin/streptavidin or digoxigenin/anti-digoxigenin) ruptures.

At the level of the polymer, it is hypothesized that the replicative (DNA polymerase), transcriptional (RNA polymerase) and translational (ribosomes) machinery responds to tension as a signal. Trivially, each of these molecular machines has a stall force which can be measured by applying a force opposite the polarity (direction of travel) of the machine. These can also stall as they encounter structures with a certain stability, such as nucleic acid hairpins and pseudoknots.

Interestingly, ribosomes coordinate as trains of ribosomes creating a protein from the same transcript. These do exhibit traffic dynamics, which can be transmitted through both occlusion (*i*.*e*., blocking of forward motion by an ahead ribosome), but also long-range interactions are possible, which are potentially mediated by DNA tension. It is known that at the nano-level, *i*.*e*., in the ribosomal entry tunnel, tension plays a large role in processes such as ribosomal frameshifting.

This nanoscale tension that occurs is qualitatively different than the tension that occurs in say, cell division, where stretches of thousands to hundreds of thousands of base pairs are pulled. In the case of nano tension in programmed ribosomal frameshifting, the tension occurs over sub-nanometer length scales (Bao *et al*. 2022).

The length scales of much of the tension that DNA experiences are on the order of the length scales between the domains of proteins, given these proteins act on DNA.

#### Tensioned amino acid chains (proteins)

**Figure 2:**
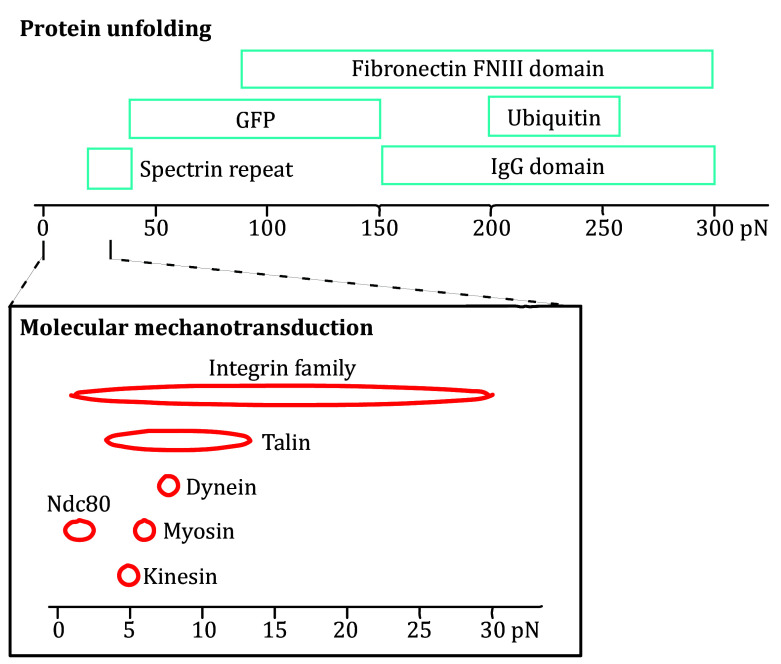
Biologically relevant force ranges for protein unfolding. Reproduced from Cost *et al*. (2015) under the terms of the Creative Commons CC BY License (https://creativecommons.org/licenses/)

Optical tweezers have proven to be an instrumental tool in studying the structural attributes and folding mechanisms of proteins, another polymer chain if we consider it at the secondary structure level, under tension. Proteins unfold over a wide variety of forces (Fig. 2) (Cost *et al*. 2015). The precise control of mechanical force provided by optical tweezers is pertinent to understanding cellular protein folding and function, allowing for the extraction of critical kinetic and thermodynamic information (Bustamante *et al*. 2020).

One of the early groundbreaking studies involved the manipulation of the giant muscle protein, titin (Kellermayer *et al*. 1997; Tskhovrebova *et al*. 1997). Comprising nearly 250 immunoglobulin-like and fibronectin domains with an approximate molecular weight of 3 MDa, titin was ideally structured for tethering between two beads within an optical tweezer’s setup, or between the tip of an atomic force microscope cantilever and a surface. This study was pioneering as it enabled the investigation of a protein's reaction to mechanical denaturation.

Optical tweezers are particularly adept at analyzing the folding pathways of proteins. Ensemble techniques struggle to clarify the energy landscape of protein folding, which involves complex diffusion over a one-dimensional trajectory. Optical tweezers overcome this challenge through force spectroscopy experiments, allowing researchers to reconstruct complete energy landscapes by employing the end-to-end extension of the protein as a well-defined reaction coordinate (Bustamante *et al*. 2004).

Additionally, optical tweezers have provided valuable insights into co-translational folding, where proteins start folding as they are synthesized by ribosomes. Due to the slow rate of polypeptide synthesis relative to rapid folding timescales, it is believed that synthesis and folding are coupled processes. Optical tweezers have been instrumental in studying the mechanisms of this coupling, as demonstrated by the investigation of T4 lysozyme folding on the ribosome (Kaiser *et al*. 2011).

Furthermore, in the complex and crowded environment within cells, molecular chaperones are essential for efficient protein folding. Optical tweezers have been used to study the role of these chaperones in guiding the folding pathways of proteins (Liu *et al*. 2019).

#### Stretching chromosomes

Chromosome condensation, separation, and transportation to the spindle poles during mitosis are fundamentally biomechanical processes. Chromosome condensation involves DNA compression, separation necessitates topological disentanglement of sister chromatids, and transportation is achieved during anaphase. These dynamic processes require forces that are either generated exogenously by the mitotic spindle or endogenously through protein-protein and protein-DNA interactions and molecular motors. However, the forces involved in mitosis and the mechanical properties of chromosomes, particularly their elastic behavior, are not well-understood, and are crucial for gaining deeper insights into chromosome structure.

Optical trapping and manipulation present an effective approach to studying the organization of human chromosomes (Meijering *et al*. 2022; Witt *et al*. 2023). This methodology facilitates high-resolution force measurements and fluorescence visualization of native metaphase chromosomes under controlled experimental conditions. Through this approach, various aspects of chromosome mechanics and structure have been investigated. One noteworthy observation is that chromosomes exhibit nonlinear stiffening behavior under increasing mechanical load. This behavior deviates from predictions made by classical polymer models. To account for this anomalous stiffening, a hierarchical worm-like chain model has been proposed, portraying the chromosome as a heterogeneous assembly of nonlinear worm-like chains.

### Molecular motors on tensioned substrates

Molecular motors operate through reactions that inherently generate force and torque. Consequently, externally applied forces and torques can influence these reactions in terms of rate, extent, or outcome, and provide insights into the dynamics and operational mechanisms of molecular motors. Here we focus on the motors involving the central dogma, replication, transcription, and translation.

#### Tension in replication

When studying DNA replication by polymerases, tension applied to DNA acts as a critical parameter. For example, by anchoring a partially ssDNA/dsDNA DNA construct between two beads and applying a force above 7 pN, as ssDNA transforms into double-stranded DNA (dsDNA) during replication, the molecule's end-to-end distance decreases. This change serves as a gauge of the polymerase's activity. However, when forces surpass 35 pN, the opposite happens; the distance increases due to the enzyme's exonuclease activity that converts dsDNA back into ssDNA, which is a consequence of tension-induced changes in the structure of dsDNA at the enzyme's active site (Wuite *et al*. 2000). Further high-resolution optical tweezers have revealed complex, irregular dynamics in DNA replication by applying tension on the DNA construct mimicking the protein-binding induced DNA tension (Hoekstra *et al*. 2017). Through a kinetic model integrating these findings with biochemical data, it was discerned that a critical error-correction pathway involving DNA polymerase ensures high-fidelity replication by extensively proofreading and removing bases.

#### Tension in transcription

The impact of force on biological motor activity was first explored in studies on T7 RNA polymerase (Yin *et al*. 1995). The force-dependent binding dynamics of several transcription factors have been quantified. Although the force applied typically stays within low piconewton (pN) ranges and thus might not seem like a significant biological stimulus, it can induce changes in DNA orientation without significantly altering its molecular conformation over short distance scales (*e*.*g*., transitioning from B- to S-form).

Under low tension, force effects are expected to manifest over longer length scales, transitioning to shorter length scales under higher tension. This correlates with the type of deformations induced by tension in these force regimes. For instance, with the single-stranded DNA binding protein RPA, there is a strong dependence on force in the low force regime, since the system is more characterized by DNA alignment rather than tension.

#### Tension in translation

Recent studies have explored the biological implications of tension in translation, such as programmed ribosomal frameshifting (Halma *et al*. 2019). For instance, pseudoknot structures can create tension on the translating ribosome, leading to stalling. Additionally, in prokaryotes, translation can simultaneously occur with mRNA transcription, a process termed coupling. Through reconstitution of the *E. coli* coupling system, it was observed that the ribosome can enhance transcription rates while compromising fidelity, by preventing RNAP pausing and termination (Wee *et al*. 2023). This shows how tension plays a critical role in modulating the interplay between translation and transcription.

### Regulation of binding patterns on tensioned substrates

In tension regulation of binding patterns, researchers use single-molecule experiments to analyze how tension affects the binding of various molecules, from small intercalators to larger complexes, to DNA. For example, as tension increases, intercalators show an increased rate of binding and a decreased rate of unbinding, likely because the space between DNA bases expands under tension, allowing easier access for these molecules (Biebricher *et al*. 2015). On the other hand, the single-stranded DNA binding protein T7 gp2.5 exhibits a strong dependence on force for binding in low tension regimes, where DNA alignment is more prominent (Xu *et al*. 2023a).

In addition to examining binding rates, these mechanical experiments enable the investigation of thermodynamics, stoichiometry, kinetics, and diffusion constants under different conditions such as ionic concentration, tension, temperature, and pH (Xu *et al*. 2023b). By understanding how SSB proteins interact with DNA and other proteins, and assessing their binding footprints, scientists can gain a deeper understanding of the mechanisms involved. Moreover, the experiments shed light on the thermodynamics of binding; for instance, high tension typically leads to the detachment of proteins from DNA. Varying salt concentrations in these experiments help researchers understand electrostatic interactions, revealing that bivalent ions are more effective in shielding DNA’s negative charges than monovalent ions. This information is crucial for understanding DNA condensation and looping mechanics.

## OTHER APPLICATIONS OF BIOLOGICAL TENSION

During cell division, microtubules play a pivotal role in pulling DNA between the centromeres of the mother and daughter cells, generating forces in the range of 2–5 pN individually (Dogterom and Yurke 1997) and tens of pN when bundled (Laan *et al*. 2008). This tension is critical for the successful separation of sister chromatids. By extending the concept of applying tension to biopolymers, scientists have garnered invaluable insights into the structure and organization of complex biological systems, such as chromatin (Brower-Toland *et al*. 2002), chromosomes ( Meijering *et al*. 2022), and the nuclei (Bergamaschi *et al*. 2019), as well as microtubules (Siahaan *et al*. 2022), through the combination of tension measurements and fluorescence microscopy. Furthermore, there is immense potential in broadening the scope of these studies to encompass other intricate organelles like mitochondria, endoplasmic reticulum, and the Golgi apparatus, which are instrumental in cellular processes including energy metabolism, protein folding, and transport. Monitoring the structural responses of these organelles to tension, while employing fluorescence labels, offers a promising avenue to deepen our understanding of their structural organization and functionality. Consequently, this knowledge could unravel new insights into the regulation of cellular processes, with implications for human health and disease.

Furthermore, there is a need to bridge the gap of tension measured between *in vitro* and *in vivo* experiments to gain a deeper understanding of molecular processes. The development of genetically encoded strategies for the measurement of forces and stresses inside the cell and at the single-molecule level is a crucial step in advancing our understanding of the role of forces in biological processes. To this end, strategies can be developed by incorporating *in* *vivo* force sensors, such as short well-characterized peptides (Ren *et al*. 2023), into biological processes such as chromosome segmentation. The application of genetically encoded strategies has the potential to provide a new level of detail in our understanding of cellular processes, as it allows for the measurement of forces and stresses in real-time and *in situ*. Furthermore, these strategies can also be used to test the effects of external stimuli, such as mechanical loads, on cellular processes. For example, scientists have been able to act physically on chromosomes in living cells (Keizer *et al*. 2022), by subjecting the chromosomes to different forces using magnets. This information can be useful in the development of new therapeutic strategies for various diseases, including cancer and neurodegenerative diseases, where mechanical forces (may) play a crucial role.

## DEVELOPMENTS IN SINGLE-MOLECULE FORCE MEASUREMENT

This field has been characterized by a significant degree of technological advancement, both in the ubiquity and ease of the systems as well as their capabilities to perform more complex experiments.

Combined force and fluorescence is a major development, allowing the simultaneous measurement of substrate (usually DNA or RNA) tension while observing the location of fluorescently tagged proteins. This is useful in determining the binding modes of single-stranded binding proteins, where the fluorescent density can be used to estimate the number of bound proteins, and the total change in extension can be divided by the number of proteins to determine the level of shortening per bound molecule (Xu *et al*. 2023a). This can also be performed at different tensions to elucidate the force dependence of protein binding (Xu *et* *al*. 2023b).

Methods have achieved greater sophistication in resolving the coordination dynamics of multiprotein complexes (Schaich *et al*. 2023). Another development is the adaptation of OT towards measuring systems *in vitro* or *in vivo*, which better recapitulates any possible post-translational modifications that a protein may experience (Clarke *et al*. 2008; Jun *et al*. 2014; Schaich *et al*. 2023).

Further developments in optical tweezers will include developments in workflows and using force measurements as a proxy measurement in drug discovery (Halma *et al*. 2022).

## CONCLUDING REMARKS

By applying and measuring forces precisely at the single-molecule level, scientists in the past decades have been able to deepen their understanding of theoretical concepts like force, torque, and displacements in chemical and biochemical reactions. Single-molecule force spectroscopy has revolutionized the way chemical and biochemical processes are viewed, framing them as mechanochemical phenomena that can be studied through externally applied forces and torques. As the product of force and displacement represents work done, it establishes a connection between single-molecule experiments and traditional bulk experiments, bridging the gap between these approaches and enhancing the understanding of molecular interactions.

Understanding tension and how it operates in various cellular processes is a significant breakthrough in the study of molecular biology. By integrating biophysical measurements like force and energy into traditional biochemical approaches, which typically focus on concentrations of reactants and products, new avenues are opened, especially in studying dynamic enzymes. This approach helps to associate structural domains with movements observed in enzymes, allowing for the reconstruction of enzyme motions at the single-molecule level. These insights can lead to the creation of novel enzymes for unprecedented reactions in biology and chemistry, much like understanding the mechanics of a motor. There are potential applications in synthetic biology, nanotechnology, and biomimicry for designing new catalysts and materials, and in medicine for developing therapies that target enzyme mechanisms.

## Conflict of interest

Matthew T. J. Halma and Longfu Xu declare that they have no conflict of interest.
